# What Do Immigrants From Various Cultures Think Is the Best Way to Cope With Depression? Introducing the Cross-Cultural Coping Inventory

**DOI:** 10.3389/fpsyg.2020.01599

**Published:** 2020-07-14

**Authors:** Valeria Markova, Gro M. Sandal, Eugene Guribye

**Affiliations:** ^1^Department of Pulmonology, Haukeland University Hospital, Bergen, Norway; ^2^Department of Psychosocial Science, University of Bergen, Bergen, Norway; ^3^NORCE Norwegian Research Centre, Agder, Norway

**Keywords:** coping, coping inventory, depression, immigrant, acculturation, Russian, Somali, Polish

## Abstract

The aim of this study is to introduce a domain-specific instrument, the Cross-Cultural Depression Coping Inventory (CCD-CI), to assess ways in which people from different cultures prefer to cope with depression. Part 1 of this paper describes the development of CCD-CI. A combined etic and emic approach in generating items was used. Principal component analysis on data from a heterogeneous sample of immigrants (*N* = 458) supported a three-factor solution labeled: Engagement, disengagement, and spiritual coping. In Part 2 confirmatory factor analysis were conducted to test if the factors replicated in a mixed ethnic sample of immigrants from Russia (*n* = 164), Poland (*n* = 127), Pakistan (*n* = 128), Somalia (*n* = 114), and Norwegian students (*n* = 248). The three-factor model fits the data well and differentiated between the ethnic groups. Most significantly, Somali followed by the Pakistani immigrants scored higher on disengagement and spiritual coping. Inspection of item-level differences showed the largest ethnic variations in coping behavior of communal or social nature. The CCD-CI factors were validated against the Vancouver Index of Acculturation. Adoption to majority culture correlated positively with engagement and negatively with spiritual Coping. Maintenance of origin culture was positively associated with both spiritual coping and disengagement. In Part 3 the construct validity of the CCD-CI was tested in relation to the Brief Cope. The magnitude of the correlations was small to moderate. Taken together results indicate that CCD-CI is a reliable and valid measure of coping strategies related to depression, suitable for adults from different ethnic groups. Implications for research and clinical practice are discussed.

## Introduction

According to the World Health Organization (2017), depression is the single largest contributor to global disability. While depression is common in all parts of the population, research shows that the prevalence is higher among immigrants from low-income countries and refugees when compared to the general population (Lindert et al., 2009; Kale and Hjelde, 2017). However, previous studies have shown ethnic differences in the use of public health services (Straiton et al., 2017) and that some immigrant groups are less frequently referred to mental health specialists than natives (Jensen et al., 2013). Research is sparse on how different immigrant groups prefer to cope with mental health problems including depression, and how coping preferences may be related to immigrants’ acculturation orientations (Kuo, 2014; Kale and Hjelde, 2017). There is currently a lack of adequate methods to further advance the research in this field; especially culture-sensitive, domain-specific coping inventories are missing (Kato, 2015). The present study aims to contribute to filling this methodological gap by introducing a new instrument designed to capture ethnic differences in views about efficient depression coping strategies. A better understanding of the roles of culture in the process of coping with depression can lead to better integration of cultural specificities into assessment, counseling, and educational activity.

In the present study *coping* is understood as constantly changing cognitive and behavioral efforts to manage specific internal or/and external demands that are appreciated as a stressor (Lazarus, 1999). Depression has been viewed as a mental condition of helplessness and hopelessness due to lack of perceived ability to cope (Levine and Ursin, 1991). However, depression is an adverse condition that people may try to overcome in different ways (Lucock et al., 2007), for example, by help-seeking from various sources, social isolation, taking medications, or cognitive reformulation. Strategies differ in efficiency, and some may even exacerbate the disease (Aldao et al., 2010).

During the past decades, several classifications of coping strategies have been suggested on theoretical grounds (Skinner et al., 2003; Kato, 2015). Searching for the structure of coping, Skinner et al. (2003) identified 400 ways of coping and recommended not to use the most common classifications of coping, such as problem- vs. emotion-focused coping proposed by Lazarus and Folkman (1984). According to Skinner et al. (2003), those classifications do not take into consideration that any given way of coping is likely to serve many functions and that all ways of coping are multidimensional. Current literature on coping also points out limitations on how coping strategies are measured. Frequently used coping scales such as the COPE (Carver et al., 1989) and Ways of Coping Questionnaire (WCQ) (Folkman and Lazarus, 1988) are broad and general, and often not suitable to assess responses to specific stressors such as depression (Ben-Porath et al., 1991; Kato, 2015). For example, one study reported that about 20 % (range: 2.1-83.9%) of the WCQ items did not apply to the stressors described by the individual participant (Ben-Porath et al., 1991). Another limitation is that coping scales tend to overlook the cultural context in which coping occurs (Kuo, 2011, 2014; Hagmayer and Engelmann, 2014).

While some aspects of depression might be universal, a growing body of research suggests that cultural differences exist in how people interpret and choose to cope with depression (Erdal et al., 2011; Hagmayer and Engelmann, 2014). For instance, a study among the Ganda in Uganda (2006) found that when witchcraft was suspected as the cause of depression, help from traditional sources and spiritual coping was sought, while Western medicine was preferred when the depression was attributed to somatic causes. In recent years several scholars have tried to take this into account and adapt domain-general coping scales like COPE and WCQ to various ethnic populations and cultural settings (Kuo et al., 2006; Wong et al., 2010; Kasi et al., 2012; Cobb et al., 2016). However, the problem persists, those measures also include items that are not relevant for certain types of stressful episodes such as depression (Rohde et al., 1990; Kato, 2015).

In this paper, we introduce a new instrument, the Cross-Cultural Depression Coping Inventory (CCD-CI). The CCD-CI was developed to offer a culturally sensitive instrument that could be used to add to our understanding of how different immigrant groups prefer to manage depression. The term ‘immigrant’ in this study refers to a person who either has immigrated to Norway or who is Norwegian born with two immigrant parents. The paper consists of three parts. In Part one, we describe how CCD-CI was developed and examine the dimensionality of the instrument by principal factor analysis on data from a heterogeneous sample of immigrants. In Part two, we validate the dimensions by using confirmatory analysis and examine the ability of factors in CCD-CI to differentiate between immigrant groups. We also examine possible relationships between the CCD-CI and acculturation orientations. Part 3 examines the construct validity of the CCD-CI with Brief Cope (Carver, 1997) as this is one of the more frequently used measures of coping.

As in previous studies on coping preferences (Kuo, 2011), our approach is based on the understanding that scientific knowledge is culturally situated, which implies that knowledge is dynamic and must be interpreted in relation to society and the context in which it is created.

Our study focuses on the belief of laypeople rather than a clinical population. Laypeople refer to persons who do not have professional knowledge of mental health treatment and disorders. The high prevalence of depression among the general population and specifically among the migrant population suggests that a large proportion will either experience depression themselves or must cope with members of their close network who experience it (Kale and Hjelde, 2017; Straiton et al., 2017). Research indicates that the social network has a strong influence on mental health service utilization and choice of coping strategies (Kuo et al., 2006; Hagmayer and Engelmann, 2014); thus the view of laypeople may be highly informative about how people experience and deal with depression.

## Part 1: Development of the Cross-Cultural Depression Coping Inventory

### Approach

The CCD-CI was developed using a combined emic and etic approach. The *emic* approach strives to describe a particular culture in its own terms, whereas an *etic* approach attempts to describe differences across cultures in terms of general, external standards (Berry, 1990).

We started with an etic approach, building on an instrument previously used in a small-scale study among immigrants in Norway by Erdal et al. (2011). The instrument included a vignette describing a depressed person and follow-up questions about appropriate coping behavior (Erdal et al., 2011). Focusing on a fictive person can be beneficial when addressing sensitive topics where the respondents may feel uncomfortable referring to their personal experiences and may reduce bias from social desirability. Further, this approach gives the possibility to examine different groups’ interpretations of a “uniform” situation and minimizes the effects of cultural and linguistic differences (Peng et al., 1997; Evans et al., 2015). In the CCD–CI, a slightly modified version of the vignette developed by Erdal et al. (2011) was used to cover the diagnostic criteria for depression in the International Classification of Diseases-10 (ICD-10) (World Health Organization, 2011). Out of 20 items in the original instrument, nine items were retained.

In the second step, we applied an emic approach. To avoid an ethnocentric bias in coping behaviors, researchers from several disciplines (anthropology, social work, psychology) and laypeople from many countries reviewed the items and were invited to suggest additional items to cover coping behavior that could be relevant in different cultural contexts. We also attempted to identify missing themes by reviewing frequently used coping instruments, including Ways of Coping Questionnaire (Folkman and Lazarus, 1988), COPE (Carver et al., 1989), and Utrecht Coping List (Schreuers et al., 1993).

The next step involved eliminating items with ambiguous content. Cultural brokers from different continents (immigrants who were familiar with both Norwegian and heritage cultures) were invited to review the items in terms of relevance and language accuracy. Finally, the selected pool of items was reviewed by a panel of researchers to reduce overlapping items, to ensure that a large range of coping behavior was covered, and to improve clarity of wording and face validity. The final version of CCD-CI used in this study consists of 28 items describing different ways that the vignette character could attempt to deal with his or her condition.

Items reflecting help-seeking and social support are not included in the current version of the CCD-CI. In a separate study (Markova and Sandal, 2016), we used an adapted version of the General Help-Seeking Questionnaire (GHSQ, Wilson et al., 2007) to examine preferences for professional, semi-professional, and informal sources of help among part of the Somali sample included in this study. While help-seeking (including social support) represents one aspect of coping that can be addressed by established measures (e.g., GHSQ), coping refers to a much wider range of behavior that may vary across cultures which may be lacking from existing measures. In the development of the CCD-CI, we were particularly interested in covering the latter group of coping behavior.

### Method

#### Participants

The dimensionality of the CCD-CI was tested on a heterogeneous sample of 458 immigrants. A total of 60.5% were females. The mean age was 36 (*SD* = 9,28). A total of 115 countries of origin were represented in the sample; 19,1% were from Africa and the Middle East, 16,7% were from Asia, 55,5 % were from different European countries and 8,7% were from America.

#### Measures

The first part of the survey consisted of questions about demographics, including age, and gender. Then *the CCD-CI* followed. Respondents were first asked to read the vignette. The vignette character was gender-matched to the respondent to facilitate identification. The vignette was as follows:

“John/Ann is a 27-year-old waiter in a restaurant in Bergen. He/she was born in Oslo to parents who were restaurant owners but has made Bergen his/her home for 5 years. In the last few weeks, he/she has been experiencing feelings of sadness every day. John/Ann’s sadness has been continuous, and he/she cannot attribute it to any specific event or to the season. It is hard for him/her to go to work every day; he/she used to enjoy the company of his/her co-workers and working at the restaurant, but now he/she cannot find any pleasure in this. In fact, John/Ann has little interest in most activities that he/she once enjoyed. He/she is not married and lives alone, near his/her brother/sister. Usually, they enjoy going out together and with friends. But now, he/she does not enjoy this anymore. John/Ann feels very guilty about feeling so sad and feels that he/she has let down his/her brother/sister and friends. He/she has tried to change his/her work habits and start new hobbies to become motivated again, but he/she cannot concentrate on these tasks. Even his/her brother/sister has now commented that John/Ann gets distracted too easily and cannot make decisions. Since these problems began, John/Ann has been sleeping poorly every night; he/she has trouble falling asleep and often wakes up during the night. A few nights ago, as he/she lay awake, trying to fall asleep, John/Ann began to cry because he/she felt so helpless.”

After reading the vignette the respondents were asked to indicate their agreement with the 28 items on a 6-point Likert scale (1: *strongly disagree*, 6: *strongly agree*).

#### Procedure

The Regional Committee for Medical and Health Research Ethics (2013/2181) and the Norwegian Social Science Data Services (36142) approved study 1 and 2.

A convenient sample of immigrants was recruited from mailing lists available on employees at the University of Bergen and from several municipalities in the Western part of Norway. The invitation was sent by mail directly to the recipient’s email address. A link to the survey was part of the invitation letter. The recipients had two weeks to answer the survey, they received only one invitation, and no reward was offered. Along with the invitation to participate in the study, recipients were informed that the study aimed to learn more about how people from different cultures think that one should best deal with feelings such as sadness and that such knowledge could inform the development of health services adapted to the needs of minority groups. They were guaranteed anonymity and informed that results would be used exclusively for research and education purposes. The recipients could choose to answer the survey in Norwegian or/and English.

#### Analysis

IBM SPSS (*Version 24.0*) was used for the statistical analyses. A principal component analysis with Varimax rotation of all items in the CCD-CI was conducted to extract coping strategies that tend to be used simultaneously. Items with loadings below 0.40 or cross-loadings of 0.40 or higher on two or more factors were removed (Howard, 2016). In addition, a modified parallel analysis was conducted to establish a number of factors. Internal consistencies were calculated with Cronbachs’ alpha.

### Results

#### Factor Structure of the CCD-CI

A principal component analysis yielded eight factors with eigenvalues exceeding one, accounting for 56% of the total variance. A scree plot and parallel analysis supported a 4-factor solution, accounting for 40% of the total variance. Three items were deleted due to cross-loadings (“*John/Ann should express his/her emotions*,” “*John/Ann should engage in leisure time activities to keep his/her mind off the situation*” and “*John/Ann should get married”)*. Five items (“*John/Ann should be ashamed*,” “*John/Ann should get more rest*,” “*John/Ann should use medication*,” “*John/Ann needs to reassess his/her life situation*,” “*John/Ann should stay at home and not work until he/she gets better*”) were deleted because of low factor loadings. In addition, one item was deleted because the content diverged from the other items with high loading on the Engagement Coping factor (“*John/Ann should get at pet*”). Thus, the final version of the CCD-CI used in the subsequent analysis consisted of 19 items. Bartlett’s test of sphericity was significant, and the Kaiser-Mayer-Olkin measure of sampling was acceptable (0.77). The final 4-factor solution accounted for 47% of the total variance (Table 1).

**TABLE 1:** Factor loadings for parallel principal component analysis with varimax rotation of coping questionnaire (*N* = 458).

	Engagement	Disengagement	Spiritual	Avoidance
.. start practicing yoga or meditate	**0.73**	−0.08	−0.04	0.19
.. get help to reconsider his/her diet	**0.65**	−0.07	0.15	0.21
.. spend more time in nature	**0.61**	0.11	−0.12	−0.18
.. get more physical exercise	**0.61**	0.04	−0.12	−0.18
.. should start using herbs and natural remedies	**0.52**	0.01	0.20	0.33
.. take some time to reflect on his/her life	**0.50**	0.04	0.15	−0.33
.. should talk courage into him/herself	**0.42**	0.38	0.08	−0.24
.. no reason to be sad	−0.11	**0.69**	−0.02	0.11
There is nothing wrong with John/Ann	−0.06	**0.61**	−0.08	0.28
.. does not need to do anything, it is just something that will go away by itself	−0.17	**0.60**	−0.05	0.29
.. keep himself/herself busy with work	0.16	**0.59**	0.22	0.05
.. avoid thinking too much	0.12	**0.56**	0.30	−0.07
.. should find a partner	0.25	**0.50**	0.23	−0.16
.. to reconcile himself/herself with God	0.07	0.13	**0.88**	0.03
.. pray or get someone to pray for him/her	0.03	0.06	**85**	0.03
.. get help to find out if he/she is a victim of malevolent witchcraft or evil spirits	−0.05	0.14	**0.63**	0.34
.. use some alcohol or other drugs (for example khat or marijuana) to become more relaxed	0.01	0.05	−0.06	**0.64**
.. not tell anyone about his/her feelings	0.01	0.31	0.13	**0.52**
.. blame someone else	−0.00	0.06	0.30	**0.50**

*Loadings ≥ 0.40 are printed in bold.*

The first-factor, explaining 13% of the variance was labeled *engagement coping.* The items with high loadings refer to direct actions to increase psychological and physical resilience such as physical activity, spending time in nature and yoga/meditation and positive self-talk. The second-factor, labeled *disengagement coping* refers to attempts to physically or emotionally separate oneself from the depressive thoughts, avoiding thinking too much, and keeping oneself busy with work. This factor also explained 13% of the variance. The third-factor, labeled *spiritual coping* referred to reconciliation with God, prayer and getting someone else to pray for him/her, and getting help to find out if (s)he is a victim of malevolent witchcraft or evil spirits. This factor explained 12% of the variance. The fourth factor, labeled *avoidance coping*, comprised blaming someone else, using drugs or alcohol, and keeping one‘s feelings to oneself, and explained 9% of the variance.

#### Reliability Analysis

Cronbach’s alpha values for the three factors engagement, disengagement, and spiritual coping were acceptable (α = 0.70, 0.68, and 0.78 respectively), but not for the fourth factor (avoidance coping, α = 0.41). The latter factor was therefore excluded from further analysis.

### Discussion

The first part of this paper examined the dimensionality of the CCD-CI. The principal-component analysis resulted in four factors, explaining 47% of the total variance, labeled *engagement*, *disengagement*, *spiritual* and *avoidance* coping, the latter factor is excluded from further analysis due to low internal consistency. The engagement factor includes several items reflecting lifestyle changes associated with psychological resilience, and proven efficient in alleviating symptoms of depression, including physical activities, meditation, and yoga (Pascoe and Bauer, 2015; Schuch et al., 2016). The disengagement factor includes items associated with mentally diverting from the depressive symptoms, including aspects of denial. Interestingly, the item “*should find a partner*” loaded on this factor. In a qualitative study among Somali refugees (Markova and Sandal, 2016) finding a spouse was emphasized to alleviate depression as this could give them other matters of concerns (e.g., children) and someone to lean on for emotional support. This interpretation fits with the content of the other items in the factor. Finally, our findings support the view that spirituality adds a distinctive dimension to the coping process in line with other studies (Pargament, 2011; Kato, 2015). As we will later discuss (in part 5.3), the items included in this factor are the result of our emic approach and go beyond items related to religion in other subscales such as brief COPE and the Cross-Cultural Coping Scale developed by (Kuo et al., 2006).

## Part 2: Confirmatory Factor Analysis and Validation

### Introduction

The second part of this paper attempted to replicate the CCD-CI factor structure with a CFA on data obtained from different immigrant groups to Norway and a Norwegian student sample. Thereafter, we examined the ability of the CCD-CI factors to differentiate between ethnic groups and the relationships between the CCD-CI factors and the immigrants’ acculturation orientations. We chose to focus on immigrants from Poland, Russia, Somalia, and Pakistan as they represented some of the largest immigrant groups in Norway at the time of the data collection. On a group level, the immigrant groups chosen are heterogeneous according to years lived in Norway and reason for migration (including both labor migrants and refugees).

#### Cultural Differences in Preferences for Coping Strategies

Earlier studies have reported cultural differences in coping preferences (Erdal et al., 2011; Kuo, 2014). For example, Erdal et al. (2011) suggested that immigrants of non-western origin differed in coping preferences in cases of depression, compared to native-born Norwegians, and that differences were particularly salient for spiritual coping. The immigrant groups included in this study differ in their religious orientation. Somali and Pakistani immigrants are among the largest Muslim immigrant groups in Norway (Østby, 2016). Earlier studies have indicated that many immigrants in these groups have become more spiritually oriented following migration (Gladden, 2012; Padela et al., 2012; Akhtar, 2014) and that they engage more in religious activities as a coping mechanism in response to stress relative to non-Muslim immigrants (Bhui et al., 2008). Research on spiritual coping among immigrants from Eastern Europe is limited (Kale and Hjelde, 2017). While both Russia and Poland are former communist nations where secular beliefs were encouraged (Massey and Higgins, 2011), the two populations seem to differ in spiritual orientation. Polish immigrants has been reported to be spiritually oriented (in their coping preferences) (Büssing et al., 2016; Guribye et al., 2018). The Russian ethnic group has been described as highly secular, becoming even more secular with migration (Massey and Higgins, 2011). The Norwegian majority population has also been described as one of the most secular, ethnic groups in the world (World Values Research, 2015).

On this background, we hypothesized that:

H1: Immigrants from Somalia and Pakistan show a stronger preference for spiritual coping than immigrants from Russia and Poland and the Norwegian student sample.

H2: Immigrants from Poland show a stronger preference for spiritual coping than immigrants from Russia and the Norwegian student sample.

### Coping and Acculturation

Acculturation and coping are interconnected; broadly, one can say that acculturation is coping with a new and unfamiliar culture (Berry, 1997; Yoon et al., 2013; Sun et al., 2016). At the group level, the acculturation process involves modifying heritage culture practices to accommodate the practices of the new majority culture (Berry, 1997). At the individual level, aspects of self-identity, including, but not limited to, attitudes, and behaviors, are adapted to adjust to the new culture’s mainstream (Ryder et al., 2000). According to Berry (1997), one does not exclude the other; migrants can maintain or neglect their home culture while simultaneously participating and acquiring values, attitudes, and behaviors related to the culture of settlement. One implication is that immigrants may keep the traditional coping preferences from their home culture (*maintenance acculturation orientation*) despite long residence time and adoption to the mainstream culture (*adoption acculturation orientation*) in many domains.

Previous research on the relationship between coping preferences and acculturation orientation suggests that engagement coping is associated with both adoption and maintenance acculturation orientation (Kuo, 2014), while preferences towards spiritual coping are negatively associated with adoption acculturation orientation, especially for Muslim immigrants (Friedman and Saroglou, 2010; Mölsa et al., 2010).

On this background, we hypothesized that:

H3: Engagement coping strategies are positively related to both adoption and maintenance acculturation orientations.

H4: Spiritual coping strategies are positively related to maintenance acculturation orientation.

### Method

#### Participants

A total of 533 immigrants from Poland, Russia, Somalia, and Pakistan settled in Norway and 248 Norwegian students took part in the study. Out of 781 responses, 79 had more than 30 percent missing data points and were excluded from all statistical analyses. Hence, the sample used in the analysis consisted of 702 respondents of Norwegian (*N* = 225, females 67%), Russian (*N* = 151, females 87%), Polish (*N* = 109, females 77%), Pakistani (*N* = 117, females 65%), and Somali (*N* = 101, females 49%) origin. The sample size was decided following a power analysis. Power analysis was conducted with G^∗^Power, version 3.0.3 (Faul et al., 2009). Setting alpha to.05 (two-tailed), power (1-β) to.80 and setting effect sizes (Cohens *d*) to 0.2 (small), 0.5 (medium) and 0.8 (large) comparing five groups shows that a total of 1200, 200 and 80 respondents were needed, respectively. As we recruited about 100 subjects from each immigrant group, we were accordingly able to detect small-to-medium and larger effect sizes.

The gender distribution differed significantly across samples, χ^2^ (4, *N* = 702) = 46.19, *p* < 0.001. The Somali respondent group was the only group with equal gender distribution; the Russian respondent group had the largest proportion of female respondents. The age of the respondents ranged from 18 to 64 years, with a mean of 30.4 (*SD* = 9.1) for the whole sample. The mean age for the subsamples ranked from 27.3 (*SD* = 7.0, Norwegian) to 34.8 (*SD* = 8.5, Russian origin). One-way analysis of variance showed that age differed significantly between the immigrant groups, *F*(4, 1861) = 25.64, *p* < 0.001. The respondents also differed regarding years of residence in Norway. The Polish immigrants had the shortest residence time (*M* = 6.1, *SD* = 5.2), with 2% being Norwegian-born to immigrant parents, followed by the Russian immigrants (*M* = 7.92, *SD* = 5.83), with 4% being Norwegian-born to immigrant parents, Somalian immigrants (*M* = 9.31, *SD* = 7.13), with 4% being Norwegian-born to immigrant parents and Pakistani immigrants (*M* = 16.70, *SD* = 8.80), with 69% of the respondents being Norwegian-born to immigrant parents.

Levels of education differed between the groups. Russian respondent group was the groups with the highest proportion of higher education (university or college); 95% of Russian respondents had some or completed higher education, the corresponding number for other groups were 80% for the Pakistani-, 79% for Polish-, and 35% for Somali respondents.

#### Measures

##### CCD-CI

The respondents answered the same version of CCD-CI as the respondents in part 1, with one exception: One item, “*John/Ann should pray or get someone to pray for him*,” was divided into two items for the Norwegian student sample; “*John/Ann should pray to God*” and “*John/Ann should ask others to pray for him.”* Because the mean and standard deviation for these two items were almost identical, 1.76 (*SD* = 1.18) and 1.66 (*SD* = 1.16) respectively, the scores of the two items were collapsed to allow comparisons between the samples.

##### The Vancouver Index of Acculturation (Paulhus, 2013) (VIA)

The Vancouver Index of Acculturation (Paulhus, 2013) (VIA): was used to measure acculturation orientation among the four immigrant groups. The inventory consists of 20 statements assessing interest and participation in one’s heritage culture (10 items, maintenance) and the mainstream (Norwegian) culture (10 items, adoption). Each item was rated on a 9-point Likert scale (1 “totally disagree” to 9 “totally agree”). The average of the ten items of each subscale was computed, providing scale scores for maintenance and adoption. Cronbachs’ alpha on data from the immigrants in this study was 0.90 for both scales. Cronbach’s alphas for the subscales in the Somali, Pakistani, Polish and Russian samples were 0.88, 0.93, 0.89, 0.88 respectively for the Maintenance subscale and 0.88, 0.90, 0.89, 0.88 for the Adoption subscale.

#### Procedures

##### Immigrant sample

The survey was distributed and collected on paper (*n* = 33) or online (*n* = 500). Only the respondents with Somali origin were offered the possibility to answer the survey on paper. The same information as described in Part 1 was presented for all respondents. After reading information about the project, respondents provided their consent by pressing the “next” button in the online version or signing a declaration of consent for those who completed the survey on paper. The respondents with Somali origin were recruited from a local Somali Café or at language school. As for the online survey, the respondents were recruited through social network sites (e.g., Facebook, online immigrant organizations). Only those who actively expressed consent received the online link or the paper version of the survey. Data were collected by the first author and four researcher-assistants with origin in Russia, Poland, Somalia, and Pakistan. Respondents with Somali and Pakistani origin could choose to answer the survey in Arabic, English, or Norwegian. Respondents with Russian and Polish origin could, in addition, choose to answer the survey in Russian and Polish, respectively. Translations were conducted using the translation-back-translation procedure, comparing versions to maximize technical, semantic, content, and conceptual equivalence.

##### Norwegian student sample

The survey was distributed online. A research assistant invited students to participate in the study via a private message on Facebook or by email. The respondents were students in higher education institutions in Norway and from different academic disciplines; 30% humanities, 30% social sciences 1% natural sciences, 16% medicine, and 13% from other disciplines.

#### Analysis

The statistical analysis comprised of five parts. First, a confirmatory factor analysis (CFA) was performed using AMOS (*Version 25.0*) to attempt to replicate the CCD-CI factor structure in a pooled mixed ethnic sample consisting of the four immigrant groups and the Norwegian student sample. Given the limited sample sizes per ethnic group, we refrained from employing confirmatory factor analysis on each of the subsamples. Second, the internal consistencies of the factors for the five ethnic groups were established. Third, Persons product-moment correlation analyses were conducted to examine the relationship between the CCD-CI factors, acculturation orientation, and background variables (only immigrants). Fourth, multivariate analysis of variance (MANOVA) with Tukey’s post-hoc tests were conducted to test whether the five ethnic groups differed in means scores on the CCD-CI factors and on individual items. Finally, hierarchical multiple regression analyses were conducted to investigate whether the CCD-CI factors explained acculturation orientation among the immigrants when controlling for gender, age and years of higher education. Age was controlled for in a separate partial correlation analysis and ANCOVA, but no significant differences were observed (results not shown).

### Results

#### Confirmatory Factor Analysis (CFA) of the CCD-CI

**FIGURE 1:**
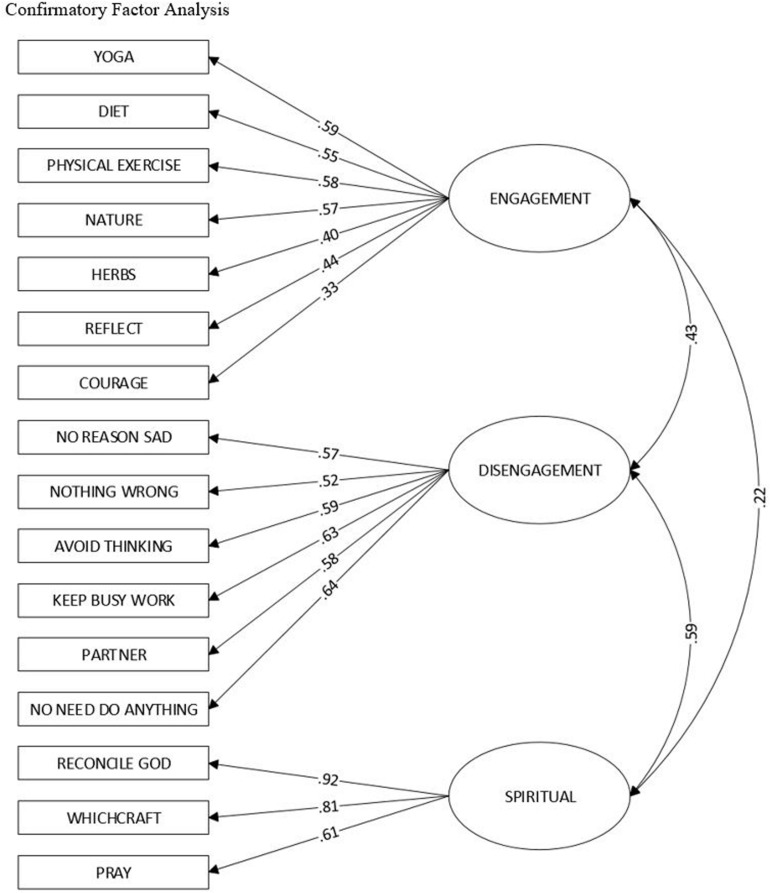
Confirmatory factor analysis.

The CFA was conducted without allowing for associations between error terms (see Figure 1). The results suggest that the three-factor model fit the data well (Brown, 2006). The chi-square to degrees of freedom ratio of 5.42 and root mean square error of approximation of 0.079 (90% confidence interval 0.073–0.086) were at the acceptable levels. However, the comparative fit index was somewhat lower (0.84) than considered optimal.

#### Reliability Analysis

##### CCD-CI

Cross-Cultural Depression Coping Inventory: as shown in Table 2, the internal consistencies of the factors were acceptable across all subsamples.

**TABLE 2:** Reliability analysis (Cronbach Alpha Coefficient) for all coping factors for all ethnic groups separately and together.

	Engagement	Disengagement	Spiritual
Somalia	0.72	0.74	0.69
Pakistan	0.68	0.73	0.70
Poland	0.66	0.76	0.74
Norway	0.75	0.73	0.77
Russia	0.68	0.80	0.71
All	0.70	0.76	0.81

#### Subscale Intercorrelation and Relations With Acculturation Orientation, Control Variables, and Background Variables

The correlational analysis (Table 3) showed that high inter-correlations between the CCD-CI coping factors. Engagement coping was positively associated with both maintenance and adoption acculturation orientation, whereas disengagement coping was positively associated only with maintenance acculturation orientation. Spiritual coping was positively associated with maintenance acculturation orientation and negatively associated with adoption acculturation orientation. Spiritual coping also correlated negatively with years of higher education. Significant gender differences were found. Females tended to score higher on engagement coping and lower on disengagement and spiritual coping then males.

**TABLE 3:** Mean, standard deviations, and correlations between coping strategies, acculturation orientation, control and demographic characteristics.

	*M*	*SD*	1	2	3	4	5	8	9
**Coping**									
1. Engagement	3.88	0.78	–						
2. Disengagement	2.81	0.97	0.37**	–					
3. Spiritual	2.26	1.34	0.22**	0.43**	–				
**Acculturation^a^**									
4. Maintenance	6.56	1.67	0.19**	0.23**	0.29**	–			
5. Adoption	5.62	1.71	0.20**	−0.01	−0.16**	0.19**	–		
**Demographic**									
8. Gender^b^	1.70	0.46	0.07*	−0.20**	−0.15**	0.02	0.05	–	
9. Age	30.44	9.10	0.06	0.03	−0.02	−0.03	−0.07	0.05	–
10. Higher Education	2.73	1.26	−0.06	−0.06	−0.18**	−0.05	0.22**	0.07	0.24**

^*a*^Only immigrant sample. ^*b*^1 = male, 2 = female. Statistically significant values marked in bold. *p < 0.05. **p < 0.01 (2-tailed).

#### Differences Across Ethnic Groups in Coping Preferences

Table 4 presents the results from the MANOVA with factor scores as dependent variables and ethnic group affiliation as the independent variable. Preliminary assumption testing checked for normality, linearity, univariate and multivariate outliers, and multicollinearity. No serious violations were noted. Levene’s test, however, showed that the assumption of the equality of variances was violated. In line with recommendations by Tabachnick and Fidell (2013), a more conservative alpha (0.025) level was therefore used. As shown by the table, scores on the three CCD-CI coping factors varied significantly between ethnic groups. The Russian immigrant group scored significantly higher than all the other ethnic groups on engagement coping, whereas the Somali followed by the Pakistani immigrant groups scored higher on disengagement and spiritual coping. As for the former result, An inspection of the magnitude of the differences between the Russian immigrant and the other ethnic groups showed small effect sizes for the Somali (*d* = 0.11) and Pakistani (*d* = 0.40) immigrant groups, and medium effect size for the Polish immigrant group (*d* = 0.40), and the Norwegian student sample (*d* = 0.62).

**TABLE 4:** MANOVA - Differences in Coping strategies based on ethnic groups (factor level).

Country of origin	Norway	Russia	Poland	Pakistan	Somalia	*F* (*4,696*)	Partial Eta Square
			
	*M* (*SD*)	*M* (*SD*)	*M* (*SD*)	*M* (*SD*)	*M* (*SD*)		
Engagement coping	3.74(0.73)_a_	**4.17(0.65)_b_**	3.88(0.78)_a_	3.87(0.77)_a_	4.08(0.95)_a_	7.641*	0.04
Disengagement coping	2.45(0.82)_*a*_	**2.97(0.98)**_b_	2.59(0.82)_a_	**3.14(0.90)**_b_	**3.24(1.16)**_b_	20.531*	0.11
Spiritual coping	1.41(0.78)_a_	**2.04(1.01)**_b_	**1.92(1.007)**_b_	**3.09(1.18)_c_**	**3.91(1.23)_d_**	128.02*	0.42

Statistically significant differences in coping strategies between ethnic groups marked in bold. Means within a row with different subscripts are significantly different at p < 0.025 *p < 0.00.

Differences between groups were also found for disengagement coping. Post-hoc tests indicated that the mean scores of respondents with Somali and Pakistani origin were significantly higher than the Polish immigrant sample and the Norwegian students with a medium effect sizes (Cohen’s *d* ranging from 0.64 too *d* = 0.79). Respondents with Russian origin were significantly higher than the Polish immigrant sample and the Norwegian student sample with small to medium effect sizes (*d* = 0.40 and *d* = 0.57).

The largest group differences were found for spiritual coping. Specifically, the Somalian respondents showed a stronger preference for spiritual coping than respondents from the other groups, and the magnitude of the differences were medium and large (Pakistani immigrant group *d* = 0.72, Russian immigrant group *d* = 1.70 and the Polish immigrant group *d* = 1.70 and Norwegian student sample *d* = 2.40). The mean score for the Pakistani immigrant sample was also significantly higher than both the Russian (*d* = 1.01) and Polish immigrant sample (*d* = 1.00), and the Norwegian student sample (*d* = 1.00) with large effect sizes. Finally, also the mean scores on spiritual coping of the Russian- and Polish immigrant samples were significantly higher than the Norwegian student sample with medium effect sizes (*d* = 0.72 and *d* = 0.60).

#### Item Level Differences Between Ethnic Groups

Table 5 shows descriptive statistics for how the different ethnic groups’ scores on individual items in the CCD-CI (including those eleven items that were taken out in earlier analysis). The table also shows results for the MANOVA and post-hoc tests. It is notable that the standard deviations of the Somali immigrants tended to be larger than for the other groups suggesting that they are more heterogeneous in views about coping behavior. Almost all items varied significantly in endorsement between the ethnic groups. Half of the items in the disengagement factor and all items in the spiritual coping factors differ in endorsement between ethnic groups with moderate to high effect size (η_*p*_^2^ = 0.06 or higher). The Somali group deviating most from the other ethnic groups. Specifically, the Somali group showed a stronger endorsement of the items *“John/Ann should find a partner”* (disengagement), and “*John/Ann should avoid thinking too much*” (disengagement). The Somali, Pakistani and Russian respondents endorsed “*John/Ann should keep himself/herself busy with work*” (disengagement) more than Norwegian and Polish respondents did. On the factor spiritual coping, the greatest group difference was found for “*John/Ann should reconcile himself with God.*” The mean score for Somali respondents was significantly higher than the mean to Norwegian respondents. There were fewer group differences on items in the engagement factor. Notably, the Russian, Polish and Somali respondents showed a stronger endorsement for the item “*John/Ann should start using herbs and natural remedies* compared to Norwegian respondents.

**TABLE 5:** MANOVA - Differences in Coping strategies based on ethnic groups (item level). Abbreviated version of questions is used in this table. For complete questions see Table 1.

Coping strategies	Norway	Russia	Poland	Pakistan	Somalia	*F* (*4,696*)	Partial Eta Square
			
	*M* (*SD*)	*M* (*SD*)	*M* (*SD*)	*M* (*SD*)	*M* (*SD*)		
**Engagement**							
.. yoga or meditation	3.57(1.34)_a_	4.23(1.32)_b_	3.90(1.35)_a,b_	3.55(1.49)_a_	3.72(1.69)_a_	6.10*	0.03
.. reconsider his/her diet	3.64(1.27)_a,b,c_	3.72(1.16)_b,c_	3.21(1.43)_a_	3.99(1.28)_*c*_	3.42(1.62)_a,b_	5.63*	0.03
.. physical exercise	4.72(1.01)_b,c_	4.91(1.04)_*c*_	4.06(1.46)_a_	4.70(1.16)_b,c_	4.44(1.53)_a,b_	9.07*	0.05
.. more time in nature	4.63(1.05)_a_	5.16(0.90)_b_	4.60(1.31)_a_	4.54(1.28)_a_	4.64(1.54)_a_	6.63*	0.04
.. herbs and Natural remedies	1.87(1.13)_a_	3.08 (1.34)_*c*_	3.04(1.52)_*c*_	2.27(1.34)_a,b_	2.51(1.61)_b_	24.20*	0.12
.. take some time to reflect	4.29(1.08_)a_	4.59(1.08)_a,b_	4.45(1.15)_a,b_	4.57(1.16)_a,b_	4.72(1.23)_b_	3.18	0.02
.. talk courage into himself/herself	3.43(1.31)_a,b_	3.47(1.33)_a,b_	3.91(1.57)_b_	3.47(1.55)_a,b_	3.03(1.78)_a_	4.59*	0.03
**Disengagement**							
.. no reason to be sad	2.00(1.16)_a_	2.68(1.39)_b_	2.50(1.39)_b_	2.60(1.35)_b_	2.66(1.79)_b_	7.96*	0.05
.. nothing wrong	2.28(1.44)_a,b_	2.65(1.36)_b_	2.09(1.42)_a_	2.79(1.47)_b_	2.27(1.65)_a,b_	4.89*	0.03
.. not need to do anything, will go away by itself	2.15(1.10)_a,b_	2.30(1.18)_b,c_	1.81(1.04)_a_	2.51(1.24)_b,c_	2.68(1.64)_*c*_	8.46*	0.05
.. busy with work	2.77(1.12)_a_	3.42(1.36)_b_	2.94(1.38)_a_	3.59(1.27)_b_	3.63(1.78)_b_	12.68*	0.08
.. avoid thinking too much	2.68(1.38)_a_	2.92(1.48)_a_	2.72(1.48)_a_	3.61(1.38)_b_	4.16(1.72)_*c*_	22.69*	0.12
.. find a partner	2.80(1.31)_a_	3.85(1.49)_b,c_	3.50(1.57)_b_	3.76(1.50)_b,c_	4.09(1.84)_*c*_	18.87*	0.10
**Spiritual**							
.. reconcile with God	1.32(0.87)_a_	2.25(1.36)_b_	2.11(1.46)_b_	3.50(1.65)_*c*_	4.76(1.51)_*d*_	134.54*	0.44
.. prey	1.79(1.26)_a_	2.46(1.54)_b_	2.27(1.48)_a,b_	3.83(1.54)_*c*_	4.42(1.60)_*d*_	76.61*	0.31
.. victim of e.g., evil spirits	1.12(0.58)_a_	1.42(1.08)_a_	1.38(0.97)_a_	1.94(1.25)_b_	2.55(1.66)_*c*_	38.57*	0.18
**Items removed**							
.. get married	1.53(1.00)_a_	2.30(1.32)_b,c_	1.94(1.35)_a,b_	2.56(1.44)_*c*_	3.60(2.11)_*d*_	40.77*	0.19
.. express his/her emotions	5.20(0.91)_*c*_	4.67(0.98)_a,b_	4.86(1.06)_a,b,c_	5.03(1.17)_b,c_	4.63(1.46)_a_	7.98*	0.04
.. leisure time activities	3.83(1.19)_a_	4.47(1.22)_b_	4.20(1.35)_a,b_	4.62(1.11)_b_	4.55(1.52)_b_	11.51*	0.06
.. use medication	2.82(1.27)_a,b_	2.93(1.27)_a,b_	3.13(1.56)_b_	2.66(1.35)_a,b_	2.58(1.80)_a_	2.65	0.02
.. should be ashamed	1.16(0.62)_a_	1.56(1.06)_b,c_	1.24(0.83)_a,b_	1.32(0.83)_a,b_	1.84(1.47)_*c*_	11.65*	0.06
.. get more rest	3.81(1.20)_a_	4.28(1.21)_b,c_	4.04(1.45)_a,b_	4.18(1.33)_a,b,c_	4.54(1.27)_*c*_	6.70*	0.04
.. reassess his/her life situation	3.36(1.36)_a_	4.23(1.24)_b,c_	3.85(1.47)_b_	3.79(1.31)_a,b_	4.43(1.41)_*c*_	14.82	0.08
.. stay at home	2.40(1.31)_a,b_	2.20(1.21)_a_	2.06(1.34)_a_	2.48(1.34)_a,b_	2.81(1.78)_b_	4.42*	0.03
.. alcohol or other drugs	1.38(0.83)_a_	1.42(0.84)_a_	1.23(0.54)_a_	1.34(0.90)_a_	1.36(1.15)_a_	0.83	0.01
.. not tell anyone about his/her feelings	1.41(1.08)_a_	2.11(1.35)_*c*_	1.50(0.96)_a,b_	1.68(1.19)_a,b_	1.88(1.54)_b,c_	8.73*	0.05
.. blame someone else	1.97(1.09)_b_	1.78(0.97)_b_	1.35(0.60)_a_	1.67(1.05)_a,b_	1.61(1.12)_a,b_	7.72*	0.04

Means within a row with different subscripts are significantly different at *p* < 0.025 *p < 0.00.

Only two of the items not included in the three-factor differed significantly between groups. with moderate to high effect sizes; “*John/Ann should get married*,” and “*John/Ann should reassess his/her life situation.*” The greatest difference was between the Somali and the Norwegian respondents with the Somali group endorsing both items more.

#### Hierarchical Multiple Regression Analysis

Finally, a hierarchical multiple regression analysis was carried out (see Table 6). Demographic variables (gender, age, and education level) were entered in the first block, followed by the three coping strategies in the second block. Missing data were handled with pairwise deletion. The results of the regression analysis showed that acculturation orientation maintenance was no longer associated with engagement coping preferences when controlling for age, gender, and education level. Preferences towards disengagement and spiritual coping explained a significant portion of the variance in maintenance acculturation orientation while preferences towards engagement and spiritual coping explained a significant portion of the variance in adoption acculturation.

**TABLE 6:** Summary of results from Hierarchical Multiple Regression Analyses.

		Maintenance (*N* = 477)				Adoption (*N* = 477)			
		*b*	*SE b*	β	*t*	*b*	*SE b*	β	*t*
Step 1:									
	Gender ^a^	−0.01	0.18	−0.00	−0.08	0.17	0.18	0.05	0.93
	Age	0.07	0.07	0.41	−0.04	−0.04	0.07	−0.21	−0.53
	Education^b^	0.07	0.07	0.42	0 1.01	−0.03	0.07	−.18	−0.47
R^2^			0.01				0.00		
Step 2									
	Gender^a^	0.28	0.18	0.08	1.56	−0.05	0.19	−0.01	−0.25
	Age	0.14	0.07	0.80	2.15	−0.08	0.07	−0.43	−1.14
	Education^b^	0.13	0.06	0.77	2.08	−0.07	0.07	−0.35	−0.94
	Engagement	0.14	0.10	0.07	1.36	0.56	0.11	0.25***	5.05
	Disengagement	0.24	0.09	0.14**	2.73	−0.08	0.09	−0.05	−0.86
	Spiritual	0.33	0.06	0.26***	5.28	−0.26	0.06	−0.21***	−4.08
R^2^Δ			0.11***				0.08***		
Total R^2^			0.12***				0.08***		

^*a*^1 = male, 2 = female; ^*b*^1 = no higher education, 5 = Ph.D. level. Statistically significant values marked in bold. *p < 0.05. **p < 0.01. ***p < 0.001.

### Discussion

In Part two, a confirmatory factor analysis was first performed to investigate factor structure proposed in part 1, the results suggest that the three-factor model fit the data well. Then we examined the ability of the CCD-CI factors to differentiate between ethnic groups and the relationships between the CCD-CI and the immigrants’ acculturation orientations.

In line with our first hypothesis, respondents with Somali and Pakistani origin prefer spiritual coping to a greater extent than other ethnic groups in this study. This is in accordance with previous research (Bhui et al., 2008; Gladden, 2012). The second hypothesis was partly supported; respondents with Polish origin prefer spiritual coping to a greater extent than the Norwegian student sample but do not significantly differ from the respondents with Russian origin.

Our third and fourth hypothesis were also partly supported. Preferences towards engagement strategies are positively associated with adoption acculturation orientation but no association was found between engagement strategies and maintenance acculturation orientation. In line with our fourth hypothesis, preferences of spiritual coping were negatively associated with adoption acculturation orientation consistent with the results of other studies (Friedman and Saroglou, 2010; Mölsa et al., 2010). One interpretation of the findings could be that spiritual coping reflects an acculturation strategy related to primarily in-group connectivity. However, the association may as well reflect the secular nature of the Norwegian society. Furthermore, the Norwegian constitution holds that the value fundament is based on Christian and Humanitarian heritage and that the King should always adhere to the Evangelic-Lutheran religion. In this framework, Muslims, Catholics, and Russian-Orthodox Christians, by necessity and not a choice, mainly need to organize their religious practices apart from the mainstream Norwegian religious institutions. Consequently, we should be cautious about our interpretations of these associations.

## Part 3. Comparison of CCD-CI With Brief COPE Introduction

Finally, we examined the CCD-CI divergent validity with the brief COPE inventory (Carver, 1997), one of the most frequently used domain-general coping instruments in the literature (Kato, 2015). This procedure allowed us to assess whether the CCD-CI scale introduces something that is not covered in already existing coping scales.

### Participants

A survey was distributed to university students. The sample consisted of 70 respondents. Ten respondents had more than 30 percent of missing data points and were excluded from further analysis. Hence, the sample consisted of 60 students (69% female). The mean age was 26 years (SD = 3.74) and the students were in different academic disciplines; 38% humanities, 10% social sciences, 30% natural sciences, and 22% from engineering degrees. The responses were anonymous and could not be linked to the individual participant.

### Measures

*CCD-CI*. The same version of CCD-CI was distributed as described in Part 1 and 2.

*The Brief Cope Inventory* (Carver, 1997) which is an abridged version of the full Cope inventory (Carver et al., 1989), was used in this study. The Brief Cope consists of 28 items and the usual instruction is to indicate agreement or disagreement on four-point scales ranging from “usually I do not do this at all” to “usually I do this a lot.” In the present study, the respondents were instructed to answer how they would act if they were in the same situation as the vignette character on a four-point scale from «not at all» to «very much». The inventory includes 14 subscales comprising two items each: (1) Active coping; (2) self-distraction; (3) using instrumental support; (4) planning; (5) using emotional support; (6) positive reframing; (7) humor; (8) religion; (9) acceptance; (10) denial; (11) venting; (12) substance use; (13) self-blame; and (14) behavioral disengagement.

### Procedure

The study was approved by the Regional Committee for Medical and Health Research Ethics and the Norwegian Social Science Data Services. The same procedure as described in Part 2 for Norwegian students were applied. The survey was distributed online via a private message on Facebook or by email by student research assistants. No reward was given.

### Analysis

First, internal consistencies (alpha coefficient) for all scales were established then Person‘s correlation analysis was conducted between CCD-CI factors and Brief Cope components (see Table 7).

**TABLE 7:** Mean, standard deviations, and correlations between coping strategies in CCD-CI and COPE (Carver et al., 1989).

	*M*	*SD*	*1*	*2*	*3*	*4*	*5*	*6*	*7*	*8*	*9*	*10*	*11*	*12*	*13*	*14*	*15*	*16*
*CCD-CI*																		
1.Engagement	3.80	0.64	–															
2. Disengagement	2.78	0.72	0.28*	–														
3. Spiritual	1.49	0.63	0.03	0.44**	–													
*COPE*																		
4. Self-distraction	2.68	0.59	−0.03	−0.21	0.09	–												
5. Active Coping	3.11	0.59	−0.03	−0.03	0.10	0.39**	–											
6. Denial Coping	1.48	0.54	0.01	0.18	−0.13	0.12	0.03	–										
7. Substance Use	1.32	0.49	0.25	0.20	0.04	0.00	−0.09	0.21	–									
8. Emotional Support	2.92	0.75	0.19	−0.28*	−0.03	0.04	0.18	0.13	−0.10	–								
9. Use of informational support	2.96	0.82	0.24	−0.26	0.01	0.14	0.32*	−0.19	−0.29*	0.61**	–							
10. Behavioral Disengagement	1.35	0.53	−0.16	0.15	0.10	−0.07	−0.19	0.23	0.25	−0.13	−0.29*	–						
11. Venting	2.45	0.60	0.09	−0.10	0.08	0.34**	0.27*	0.34**	0.20	0.41**	0.28*	0.09	–					
12. Positive reframing	2.70	0.74	0.42**	0.11	0.03	0.21	0.45**	0.14	−0.05	0.20	0.25	-0.12	0.19	–				
13. Planning	2.96	0.63	0.27*	0.04	−0.02	0.04	0.31*	0.24	0.05	0.36**	0.27*	0.17	0.19	0.44**	–			
14. Humor	2.37	0.90	0.14	0.04	−0.05	0.14	0.41**	0.12	0.13	0.04	0.20	−0.03	0.17	0.50**	0.29*	–		
15. Acceptance	2.05	0.60	0.0	0.05	−0.15	0.07	0.19	0.32*	0.06	−0.02	−0.08	0.03	−0.02	0.18	0.17	0.15	–	
16. Religion	1.51	0.72	0.30*	0.35**	0.68**	−0.04	0.29*	−0.01	0.03	0.08	0.20	0.06	0.18	0.27*	0.25	0.16	−0.09	–
17. Self-blame	2.37	0.86	−0.18	0.03	0.09	−0.10	−0.43**	0.06	0.20	−0.11	−0.20	0.34**	0.20	−0.33*	−0.07	−0.09	−0.26	−0.05

Statistically significant values marked in bold. *p < 0.05. **p < 0.01 (2-tailed).

### Results

Internal consistencies values for the three factors engagement, disengagement, and spiritual coping in CCD-CI were acceptable (α = 0.71, 0.64, and 0.80 respectively). As can be seen from Table 7, significant correlations were few and of small to moderate magnitude suggesting that the amount of shared variance between these two measures is modest. The strongest association was found between Religion (Brief COPE) and Spiritual Coping (CCD-CI). Religion also correlated positively with both engagement and disengagement. Positive reframing (COPE) and Planning (COPE) were both positively associated with engagement coping (CCD-CI).

### Discussion

The correlations between CCD-CI and Brief Cope were generally low, and the magnitude of the significant correlations were of moderate magnitude. These results suggest that the amount of shared variance between these two instruments is small. The association between religion- and spiritual coping is not unexpected, however, the two scales have some important differences. The religion subscale in Brief COPE consists of the two items “*I’ve been praying or meditating*” and “*I’ve been trying to find comfort in my religion or spiritual beliefs.*” This content is narrower than the spiritual coping subscale in CCD-CI, consisting of the three items “*..needs to reconcile himself with God*,” “..*pray or get someone to pray for him/her*,” and “..*should get help to find out if he is a victim of malevolent witchcraft or evil spirits.*” Whereas the two items in the religion subscale in Brief COPE deal with introspective individual cultural practices, two of the items in the spiritual coping subscale in CCD-CI also deal with cultural practices that are of a more communal or social nature. In many parts of the world, belief in witchcraft and evil spirits has retained its salience, being linked with ways to regulate human conduct, address interpersonal conflicts, and restore social tensions in response to modernity and social change (For an overview see; Moore and Sanders, 2001; Dein, 2016). Moreover, the item addresses the need to seek a type of external social support in response to symptoms of depression, which only makes sense in certain cultural perspectives. Similarly, unlike in the Brief COPE subscale, the item about praying in CCD-CI specifically refers to this activity as a possible communal practice (“..*pray or get someone to pray for him*”), which can also be linked with a sense of reconnecting with one’s social network as part of the coping process. Thus, the spiritual coping in CCD-CI represents a broader focus in terms of religious activities that are relevant in a culturally diverse context, and which provides us with information about the role of social networks in the recovery process. Interestingly, in our analysis meditation and praying loaded on different factors emphasizing that they represent distinct coping behaviors (engagement and spiritual coping).

Positive reframing (COPE) and planning (COPE) were moderately correlated with engagement coping (CCD-CI). While item contents show little overlap, these scales reflect an underlying theme of actively attempting to deal with the problem. However, a difference is that the former scales tend to refer to cognitive reframing and changing the situation whereas most items in the engagement coping scale involve lifestyle changes that may build resilience. Thus, our analysis support that the engagement factor presents coping behavior beyond the scales included in Brief COPE.

## General Discussion

The results of this study suggest that CCD-CI is a reliable and valid measure of coping strategies related to depression, suitable for adults from different ethnic groups. An asset of CCD-CI above many other coping scales is that it was developed through an emic approach and consequently contains a broader reference to coping behavior which makes sense in various cultural perspectives (e.g., witchcraft and evil spirits, herbs and natural remedies, shame, and getting a partner).

This kind of inventory has been sought in recent literature (Hagmayer and Engelmann, 2014; Kato, 2015). Principal factor analysis on data from a heterogeneous immigrant sample (part 1) suggested a three-factor solution representing three distinct coping strategies labeled *engagement*, *disengagement*, and *spiritual coping*. Confirmatory factor analysis based on data from five ethnic groups (part 2) supported the three-factor structure and established evidence for the ability of the factors to differentiate between coping preferences in different ethnic groups. Specifically, and in line with past research, endorsement of spiritual and disengagement coping was more common among Somali and Pakistani immigrants than among Russian and Polish immigrants and Norwegians. The three coping factors were also meaningfully related to immigrants’ acculturation preferences measured by VIA, suggesting promising validity of the CCD-CI. Finally (part 3), we examined the divergent validity of the CCD-CI with the brief COPE inventory (Carver, 1997), one of the most frequently used domain-general coping instruments in the literature. The analysis suggested that the amount of shared variance is modest indicating that the two instruments measure different aspects of coping.

An important limitation noted in past literature reviews that have reported cultural differences in coping preferences, is that coping is typically defined, categorized and measured differently from study to study, reducing comparability (Kuo, 2011; Gladden, 2012). This points towards the need for a standardized instrument to drive research in this field forward in a more coherent way. The CCD-CI represents a first step in the development of a domain-specific coping instrument that accommodates this need. Although the target groups may not necessarily understand the described symptoms like *depression* in a western bio-medical sense of the term, the instrument allows for the investigation of culturally sensitive responses to specific behavior associated with depression. Thus, the instrument addresses previously found shortcomings with coping measures that rely on broadly applicable, domain-general coping scales; overlooks the cultural context in which coping occurs; or relies on different measures that make comparison difficult (Hagmayer and Engelmann, 2014; Kato, 2015; Alemi et al., 2016).

### The Dimensionality of the CCD-CI

The three factors identified by our analyses were conceptually meaningful. Most items loaded on engagement and disengagement coping. The third-factor spiritual coping added a distinctive dimension to the coping process (Pargament, 2011; Kato, 2015). This is an important contribution as frequently used coping scales have, for a long time, been criticized for ignoring the importance of spiritual coping as a central distinctive coping strategy (Kato, 2015). In addition, the findings of the present study suggest that the three coping strategies were interrelated. This is consistent with previous studies (Tobin et al., 1989; Wong et al., 2010) and supports the view that people typically use a mixture of several types of coping strategies, which may change over time (Mölsa et al., 2010).

The Russian, Pakistani and Somali immigrant groups chose all three coping strategies to a greater extent than the Norwegian student group. This may indicate a distinctive culturally related coping style. Wong et al. (2006) argued that individuals from collectivistic or more eastern cultures could embrace paradoxical and dualistic forms of beliefs that influence coping. For example, earlier studies have shown that both Pakistani and Somali immigrant groups might simultaneously subscribe the reason for depression to culturally influenced beliefs that can be characterized as spiritual (e.g., Jinn possession) and/or situational (e.g., isolation in the new country) problems (Mölsa et al., 2010). This apparent duality makes more sense when considering that cultural practices such as witchcraft and the occult has previously been linked with ways of articulating fears about the increased uncertainties of everyday life and the global economy (Moore and Sanders, 2001). Thus, the two explanations may not be as remote from each other as they seem. The assumed multicausal nature of mental distress, may also make many different coping strategies seem appropriate. However, we cannot exclude the possibility that also response bias, such as social desirability, has led to high correlations between the coping scales.

### Engagement and Disengagement Coping

In line with previous studies (Kuo et al., 2006; Cobb et al., 2016), our results showed significant ethnic differences in preferences towards disengagement coping. Disengagement and avoidance have been interpreted as perceived insufficient resources to manage the situation (Cobb et al., 2016), and associated with depression, as well as psychopathology more generally (Aldao et al., 2010; Orzechowska et al., 2013). Conversely, engagement has been assumed to reflect positive coping expectancies and has been linked to less psychopathology and more psychological resilience (Rohde et al., 1990). However, this pattern may not persist in a cross-cultural context. Several studies have found that disengagement coping may be associated with positive psychological outcomes for some ethnic groups (Kuo et al., 2006; Cobb et al., 2016). Kuo et al. (2006) argue that preference towards disengagement strategies may sometimes be motivated by the preferences observed in many collectivistic cultures for interdependence and preservation of social harmony. Consistent with this assumption, immigrants from Russia, Somalia, and Pakistan, more collectivistic oriented cultures, were most inclined to endorse disengagement. However, as the present study did not include mental health outcome variables, the efficiency of coping strategies could not be assessed. This is one avenue for future research.

The mean score for engagement coping was high for all ethnic groups suggesting that certain coping behavior to deal with depression tend to be universally endorsed among laypeople, for example, recommending physical exercise. Importantly, while we see the same tendencies towards a distinction between engagement/disengagement coping as in previous studies, the CCD-CI allows us to investigate differences on an item level that may also be useful when dealing with ethnically mixed groups in a clinical or research contexts, e.g., Somalis are more inclined to recommend finding a partner, Russians are more inclined to recommend herbs and natural remedies.

Our hypotheses about relationships between coping and acculturation were not fully supported. In line with previous research (Friedman and Saroglou, 2010; Mölsa et al., 2010; Kuo, 2014), we expected to find that engagement coping was associated with both maintenance and adoption acculturation orientation, and that preferences towards spiritual coping was negatively associated with adoption acculturation orientation. However, we found that disengagement was associated with maintenance acculturation; engagement was associated with adoption acculturation; and spiritual coping was associated with both. These findings may reflect that both coping preferences and acculturation orientations are highly dynamic processes that influence each other in complex ways. For instance, Mölsa et al. (2010) showed that for Somalis in exile, coping strategies both persist and change as a result of the encounter with the Finnish biomedical system and to new religious interpretations by Somali religious scholars in Finland. Thus, we need more knowledge on the interplay between coping preferences and norms in both the heritage culture and the country of settlement.

### Spiritual Coping

Differences between ethnic groups were most considerable in preferences towards spiritual coping. Even though our findings support earlier findings reported by Erdal et al. (2011) who demonstrated that the differences in coping preferences towards spiritual coping are largest between non-western and western groups, our findings show that respondents from Poland and Russia also significantly differ from Norwegian respondents when it comes to spiritual coping. The result is in the lines of earlier literature (Kasi et al., 2012; Büssing et al., 2016; Guribye et al., 2018), which found that spirituality plays an important part in Polish migrant communities. Against our expectations, Russian respondents preferred spiritual coping, similar to the Polish respondents. This is interesting because it may indicate that spirituality is more important for Russian respondents than earlier reported (Massey and Higgins, 2011). Together, this adds to the literature that emphasized the need for more research on eastern European immigrants in the context of Norway (Kale and Hjelde, 2017).

Our findings indicate that respondents from Pakistan, and Somalia have a preference towards combining spiritual coping with engagement and disengagement coping, congruent with earlier research (Pargament, 2011). Earlier research by Wink et al. (2005) has shown that greater involvement in spiritual coping buffered the effects of depression associated with poor physical health, even after controlling for general social support (Wink et al., 2005). This is because spiritual coping not only provides mosque or church-based support, but also a strong and historically based sense of belonging, values, and identity. This is interesting in connection with the findings in both our study and previous studies (Okello and Ekblad, 2006; Mölsa et al., 2010) that preferences for spiritual coping were positively associated with maintenance acculturation orientation and negatively associated with adoption acculturation orientation. In other words, spiritual coping may reflect an acculturation strategy emphasizing interest and participation in one’s own heritage culture. Importantly, the items in the subscale do not only include an emphasis on cultural traditions, but also on social networks (“*I enjoy social activities with people from the same heritage culture as myself,”* and *“I am interested in having friends from my heritage culture”*). As previously discussed, the Spirituality subscale also includes references to social support (“..*pray or get someone to pray for him*,” and “..*get help to find out if he is a victim of malevolent witchcraft or evil spirits*”). Consequently, the spirituality subscale in the CCD-CI provides a way to assess important social dimensions of coping and acculturation, which may be valuable when collaborating with patients in deciding culturally appropriate treatment programs.

### Limitations and Future Studies

Even though results from the current study provide initial support for the validity and reliability of the CCD-CI, several limitations should be noted. Our findings are based on the views of laypeople about how depressive symptoms could be coped with. While laypeople may play an important role in advising depressed family members or friends, their views do not necessarily correspond to how depressed individuals themselves would act. Future studies are needed to consider whether the ethnic differences found in this study hold in clinical samples of depressed individuals. An interesting avenue for further research is to examine potential ethnic differences between health professionals. It can be questioned whether the response to how a fictive vignette character should deal with depressive symptoms correspond to how the respondents themselves would act if they or someone in their family were depressed. Previous studies have shown that participants tend to respond to hypothetical and real-life scenarios in a similar manner (Peng et al., 1997; Evans et al., 2015). Nonetheless, future research should consider comparing responses to the current version of the CCD-CI with self-report within a clinical group.

Participants were recruited primarily via Facebook and other social media. Although some research suggests that samples recruited through Facebook are as representative of the general population as samples recruited through traditional methods (Thornton et al., 2016), other studies highlight that suboptimal recruitment may occur due to mismatch between the target population and the social media platform demographic profile of users (Mellon and Prosser, 2017). Besides familiarity and use of social media platforms, low reading literacy, and lack of familiarity with questionnaires are factors likely to have prevented participation in the study. Readers should be mindful that these and other factors may influence on the comparability of the samples, for example, many respondents in the Pakistani immigrant group were of the second generation. Because the students are both younger and more educated than the immigrant groups, we cannot rule out the possibility that age or level of education–or an interaction–is responsible for the differences between the Norwegian sample and the immigrants. Future studies including more comparable ethnic samples are needed to replicate and extend the findings of this study, and test-retest reliability should be examined. We cannot exclude the possibility that social desirability and other response biases, included scale usage, to some extent influenced the results. However, we believe that the possible impact of these biases was small as there was no clear pattern in that certain ethnic groups consistently scored higher or lower on the scale- or item levels.

An asset of the CCD-CI is that individuals from a variety of cultures and different professional background were involved in assembling items. As a result, it contains a broad range of coping behavior not represented in more traditional measures such as Brief COPE. Including a clinical group with depressed patients is an important aspect for further development of the CCD-CI. Future expansion of the CCD-CI should also involve more items reflecting seeking social support as a way of coping with depression. Some items standing out as culturally sensitive are largely communal in nature, for example “*finding a partner*” and “*pray or get someone to pray for him/her.*” In a previous mixed-method study based on part of the Somali sample, the General Help-Seeking Questionnaire was included (Markova and Sandal, 2016). The results showed that many would seek advice from religious leaders and members of the ethnic community. Future developments of the CCD-CI should consider including questions about preferred help-seeking sources. Further studies should also provide more evidence on construct validity by examining possible overlap with other coping scales developed for ethnically diverse contexts, for example, the Cross-Cultural Coping Scale (Kuo et al., 2006).

### Conclusion and Implication

Taken together, the results presented in this paper suggest that CCD-CI is a culturally sensitive and valid, domain-specific coping inventory. Our results showed substantial ethnic differences on all the three coping factors, engagement, disengagement, and spiritual coping. Most notably, spiritual coping emerged as the factor with the largest variability. On an item level, ethnic differences were noted in coping behavior that are largely communal in nature and absent from established coping scales, including “getting married” and “finding a partner.” While we emphasize the need for further development and extension of the instrument, we argue that the present study suggests that the CCD-CI makes an important contribution to the coping literature. Indeed, the differences observed in this study support the need to differentiate between immigrant groups in research on how people prefer to cope with symptoms of depression, aided by a standardized instrument (Kuo et al., 2006; Kuo, 2014).

We suggest that the CCD-CI should be used as a flexible system with or without the vignette. The vignette approach may also be more suited for research purposes to provide information about coping and resilience from a cultural perspective. Thus, we gain a better understanding of what individuals in different cultural groups do to regulate their practices in a proactive/preventive perspective (Aspinwall and Taylor, 1997; Guribye et al., 2011). The culturally valid items included in the instrument can provide the starting point for therapeutic dialogs with patients in a clinical setting, ensuring that the treatment program takes into consideration the patient’s individual and cultural coping preferences. In therapeutic and consultancy contexts, the vignette could be helpful to generate conversation between health care providers and patients who may be reluctant to discuss mental health issues due to shame, taboo, or other matters. Generally, we suggest that the CCD-CI will provide clinicians with a useful tool to assess preferred ways of acquiring social support and coping with symptoms of depression among immigrant patients.

## Data Availability Statement

The datasets generated for this study are available on request to the corresponding author.

## Ethics Statement

The studies involving human participants were reviewed and approved by the Regional Committee for Medical and Health Research Ethics. The patients/participants provided their written informed consent to participate in this study.

## Author Contributions

VM led the conception and design of the study, analysis, interpretation of the data, drafting, writing, and revising the work. All authors contributed to the design, analysis and interpretation of the data, and/or writing and revising the work critically for important intellectual content and read and approved the final version of the work to be published.

## Conflict of Interest

The authors declare that the research was conducted in the absence of any commercial or financial relationships that could be construed as a potential conflict of interest.
